# Impact of increasing dietary energy on fattening steers growth performance, feed efficiency, and metabolic traits

**DOI:** 10.1007/s11250-025-04608-z

**Published:** 2025-08-05

**Authors:** Hany M. Gado, Dalia A. Ahmed, Mona M.M.Y. Elghandour, Pasquale De Palo, Angela Gabriella D’Alessandro, Abdelfattah Z. M. Salem

**Affiliations:** 1 Faculty of Agriculture, Ain Shams University, P.O. Box 68, Hadayek Shoubra, Cairo, 11241 Egypt; 2 Faculty of Agriculture, Cairo University, El-Gamaa Street/Orman - Giza, Cairo, 12613 Egypt; 3 Facultad de Medicina Veterinaria y Zootecnia, Universidad Autónoma del Estado México, Toluca, Estado de México México; 4 Dipartimento di Medicina Veterinaria, Università degli Studi di Bari, 70010 Valenzano, Bari, Italy; 5 Dipartimento di Scienze del Suolo, della Pianta e degli Alimenti (Di.S.S.P.A.), Università degli Studi di Bari, Via Giovanni Amendola, 165/a, Bari, 70126 BA Italy

**Keywords:** Beef cattle, Feed efficiency, Energy, Fermentation, Single-cell protein

## Abstract

This study aimed to evaluate the effects of a high-energy supplement (XE), as a high-energy nutritional supplement, on the growth performance and feed efficiency of fattening steers. The XE consisted of glycerol, propylene glycol, and a single-cell protein formula. Sixty Brazilian steers, with an initial body weight of 300 kg, were randomly assigned individually into three treatment groups with the addition of XE at 0% (control), 0.5% (XE1) and 1.0% (XE2) to the diet. Average daily gain, total weight gain, nitrogen balance, feed intake, and blood metabolites were measured. The daily gain was increased (*P* < 0.05) in XE1 and XE2 steers (1.13 and 1.23 kg/day, respectively) compared to control steers (1.04 kg/day), indicating improved feed efficiency due to the enhanced energy density provided by the supplement. Total weight gain showed a significant increase (*P* < 0.05) in XE2 (147.0 kg) compared to XE1 (136.5 kg) and the control steers (124.5 kg). Feed efficiency rose by about 17% (*P* < 0.05) in XE2 compared to the control group. Nitrogen intake, excretion, and retention patterns also provided insight into the effect of XE on protein utilization. Blood metabolite analysis revealed that XE supplementation enhances metabolic efficiency, nutrient utilization, and overall health in fattening. The study demonstrates that energy-dense diets can improve growth rates in fattening beef by improving feed efficiency and increasing the rate of weight gain without increasing dry matter or nutrient intake. The XE as a feed supplement can enhance the growth performance and metabolic profiles of fattening steers.

## Introduction

The growing global demand for high-quality beef has prompted improvements in beef production procedures, notably in terms of nutrition and growth efficiency (Smith et al. 2018; Pulina et al. 2021). Optimal growth rates and feed efficiency are crucial factors that directly influence the economic viability of beef production systems (Guarnido-Lopez et al. 2023). In this context, feed additives have emerged as valuable tools to enhance growth performance, improve feed conversion ratios, and promote overall animal health (Pandey et al. 2019). Probiotics are known to modulate gut microbiota, enhance digestion, and increase nutrient absorption, leading to improved growth performance (Fu et al. 2023; Liu et al. 2023). The use of probiotics can impact average daily gain and feed efficiency of fattening cattle (Dias et al. 2022; Kamalakannan and Devamugilan 2024), in addition to improved metabolic profiles, including enhanced blood parameters, which are vital for overall animal health and productivity (Ponnampalam et al. 2024). Alternative protein sources like plant byproducts, insects, and microorganisms are gaining attention due to their ability to utilize renewable feed stocks from agrifood residues without needing arable land. Single-cell protein (SCP) is a term used to describe microorganisms with high protein levels that come from bacteria, yeast, fungi, and algae. The SCP is high in vital amino acids, lipids, and vitamins, and as a protein supplement, it serves as an eco-friendly substitute for animal proteins. Global protein demand is rising, efficient SCP production relies on quick microbial growth, high yields, and diverse fermentation substrates (Spanoghe et al. 2021; Koukoumaki et al. 2024; Li et al. 2024). The inclusion of SCP in the formulation enhances feed conversion efficiency, allowing animals to gain more weight with less feed. The SCP contributes to the energy content by providing fermentable protein, leading to the production of volatile fatty acids in the rumen, a vital energy source for ruminants.

The XE, an energy supplement, has been proposed to improve the energy levels in fattening diets of animal production. It contains glycerol and propylene glycol blended through fermentation, including Single Cell Protein in the formulation. Thus, the objective of this study was to investigate the effects of the XE additive on growth performance, feed efficiency, and blood metabolites in fattening steer diets. By assessing these parameters, we aimed to provide insights into the potential benefits of incorporating XE into beef cattle nutrition, thereby contributing to more sustainable and profitable beef production practices.

## Materials and methods

This study was approved by the Care of Experimental Animal and Research Ethics Committee of Ain Shams University, Agriculture Sector Committee (Approval No. 4-2024-11). The use of animals in our study was in strict accordance with the Directions for Caring for Experimental Animals according to the American Veterinary Medical Association (AVMA). Animals were sacrificed according to the AVMA Guidelines for the Euthanasia of Animals (AVMA 2020).

### Energy product

The energy product used (XE) is a high-energy nutritional supplement for livestock, especially fattening steers, designed to improve performance and efficiency. It contains glycerol and propylene glycol blended through fermentation by *Saccharomyces cerevisiae* and Bacillus sp., which are not genetically modified, manufactured by Bactizad Co. in Cairo, Egypt. XE has an energy content of 3942 kcal/kg as metabolizable energy (ME).

### Experimental design

A total of 60 Brazilian steers with an initial body weight (BW) of approximately 300 kg in a private farm located in Noubariya (New Nubariya, a town in Egypt, approximately 160 km north of Cairo) were randomly divided into three groups. Group C (Control), fed a basal diet, Group XE1, fed the basal diet supplemented with a low dosage of XE (0.5% by weight of the diet), Group XE2, fed the basal diet supplemented with a high dosage of XE (1% by weight of the diet). Steers were fed a total mixed ration consisting of 60:40 concentrate to roughage ratio, formulated to meet NRC requirements. The trial lasted for 90 days, including a 14-day adaptation period. Steers were housed individually in covered feedlot pens equipped with automatic waterers and feed bunks. Feed mixer was used to combine the ingredients until they were consistent, and XE was added to the diets every day. Animals were fed twice a day (08:00 and 16:00) to ensure consistent intake.

### Experimental diets

The basal diet was formulated using locally available feed ingredients, including corn silage, soybean meal, and concentrate feed, with the composition being adjusted to meet the nutritional requirements of fattening cattle, as determined by the Nutrient Requirements of Beef Cattle (NRC 2016). Table 1 displays feed components, chemical composition, and nutritive values of control and experimental rations. The intake and refusal were measured individually; there were 20 animals per experimental group, housed individually in feedlot pens. The digestibility trial utilized 3 animals per experimental group. Animals were housed individually in metabolic crates equipped with fecal and urine collection systems.

### Feed chemical analysis

Samples of feed were collected weekly, stored and analyzed for nutrient content following standard procedures. Dry matter (DM), crude protein, crude fiber, fat and ash were determined based on international methods (AOAC 2005). Neutral detergent fiber, acid detergent fiber, and non-structural carbohydrates were measured using the method of Van Soest (2018). Measuring energy values such as total digestible nutrients, gross energy, digestible energy, metabolizable energy, net energy for maintenance, and net energy for gain were calculated using the NRC equations (NRC 2016).

### Animal performance

Animal body weights were recorded at the beginning of the experiment and subsequently at 30 days. The difference between the beginning and final weights was divided by the number of days in the period to determine the average daily gain (ADG). The digestibility trial utilized 3 animals per experimental group. The animals were permitted to adjust to the feed offer for 15 days, followed by a collection of feed samples for analysis, and feed intake was recorded daily on an individual basis. To assess the nutrients, the samples were crushed, dried, and examined (Khan et al. 2003).

Feed efficiency (FE) was determined as the weight gain divided by dry matter intake (DMI). Nitrogen balance and nitrogen intake were calculated from feed nitrogen content and DMI, while nitrogen excretion was measured from fecal and urinary samples collected at 30-day intervals. Nitrogen retention was estimated as the difference between nitrogen intake and excretion (Tedeschi and Fox 2020).

### Blood parameters

Blood samples were taken from the jugular veins of five animals in each group at 4 h after morning feeding and centrifuged at 20 min at 3000 rpm. The supernatant was collected, frozen, and kept at − 20 °C for further investigation. Blood serum was calorimetrically analyzed to measure (glucose, blood urea nitrogen, total protein, albumin, globulin, creatinine, calcium, phosphorus, lactate, triglycerides and cholesterol using commercial kits (Biodiagnostic, Dokki, Giza, Egypt) according to the manufacturer instructions.

### Statistical analysis

Individually collected animal information was statistically analyzed using SPSS software (2023). Data was analyzed using a completely randomized design with ANOVA to test the effect of nutritional treatments on growth performance, nitrogen balance, feed efficiency and metabolic traits. Post-hoc comparisons were performed using Tukey’s test when were detected (*p* < 0.05) to identify significant differences among the various treatments using the following model:

$$\:{Y}_{i}= \mu+T_{i}+e_{ij}$$

Where: Y_i_: the dependent variable; µ: the overall mean, T_i_: the effect of treatments; e_ij_: experimental error.

## Results

### Energy values of feed

There was a slight increase in the gross energy values in XE1 (4.25 Mcal/kg) and XE2 (4.30 Mcal/kg) steers *versus* the control (4.20 Mcal/kg). Net Energy for gain values also improved (*P* < 0.05), rising from 1.2 Mcal/kg in the C group to 1.3 and 1.4 Mcal/kg in the XE1 and XE2-energy steers, respectively (Table 2).

**Table 1:** Feed component composition, chemical composition, and nutritional values for the experimental diets^2^

Feed Component (%)	Control	XE1	XE2
Corn	40	40	40
Soybean meal	15	15	15
Wheat bran	10	10	10
Alfalfa hay	10	10	10
Corn silage	15	15	15
Vitamins & minerals^1^	5	5	5
Salt	2	2	2
Molasses	3	3	3
X-energy	-	0.5	1.0
Chemical analysis (%)			
Dry matter	87	87	87
Crude protein	15.1	15.5	16
Neutral detergent fiber	32	31.5	31
Acid detergent fiber	11	10.8	10.5
Fat	4.5	4.8	5
Ash	7.0	7.0	7.0
Calcium	0.8	0.8	0.8
Phosphorus	0.6	0.6	0.6
Sodium	0.1	0.1	0.1
Nutritive value:			
Total digestible nutrients (%)	70	72	73
Metabolizable energy (Mcal/kg)	2.69	2.75	2.80

^1^Vitamins: 48 × 10^5^ I.U (A), 6 × 10^2^ mg (B6), 20 mg (Biotin), 8 × 10^5^ I.U. (D3), 144 mg (E), 400 mg (B1), 1600 mg (B2), 4 × 103 mg (pantothenic acid), 4 mg (B12), 4 × 102 mg (niacin), 2 × 105 mg (choline chloride), and 400 mg (folic acid). Minerals: 12 × 10^3^ mg Iron, 16 × 10^3^ mg Manganese, 12 × 10^2^ mg Copper, 120 mg Iodine, 80 mg Cobalt, 40 mg Selenium, and 16 × 10^3^ mg Zinc

^2^XE = X-energy (glycerol, propylene glycol, and a single-cell protein formula); XE supplemented at 0% (control), 0.5% (XE1) and 1.0% (XE2) of the diet

**Table 2:** Distribution and values of energy for gain and maintenance among experimental diets^1^

Energy type (Mcal/kg DM)	Control)	XE1	XE2	SEM
Gross energy	4.20	4.25	4.30	0.05
Digestible energy	3.20	3.40	3.60	0.2
Metabolizable energy	2.80	3.00	3.20	0.2
Net energy for maintenance	1.70	1.80	1.90	0.1
Net energy for gain	1.20^b^	1.30^a^	1.40^a^	0.1
Total daily energy Intake	36.6	38.5	40.5	1.95
Total daily energy for maintenance	24.5	26.0	27.5	1.5
Total daily energy for gain	12.1	12.5	13.0	0.45

^1^XE = X-energy (glycerol, propylene glycol, and a single-cell protein formula); XE supplemented at 0% (control), 0.5% (XE1) and 1.0% (XE2) of the diet

Numbers with different letters (a, b,c) in each row differ significantly at P < (0.05)

### Animal weight gain

Over the 120 days of the experimental period, XE supplementation had a notable effect on overall weight gain, which made the final body weight increase (*P* < 0.05) by about 6% compared with the control group. The nitrogen balance improved (*P* < 0.05) with XE supplementation by about 10 to 21% in XE1 and XE2-energy groups *versus* the control group (Table 3).

**Table 3:** Growth performance, nitrogen balance and feed efficiency in steers across experimental groups^1^

Metric	Control	XE1	XE2	SEM	*p*-value
Initial body weight (kg)	300.0	301.5	303.0	1.5	0.120
Final body weight (kg)	424.5^c^	436.5^b^	450.0^a^	7.5	0.021
Average daily gain (kg/day)	1.04^c^	1.13^b^	1.23^a^	0.045	0.018
Total weight gain (kg)	124.5^c^	135.0^b^	147.0^a^	7.5	0.015
Average daily dry matter intake (kg/day)	16.5	16.3	16.1	0.4	0.091
Feed efficiency	0.063^b^	0.068^b^	0.074^a^	0.006	0.030
Nitrogen intake (g/day)	475^c^	495^b^	515^a^	20	0.022
Nitrogen excreted (g/day)	190^a^	180^b^	170^c^	10	0.025
Nitrogen retained (g/day)	285^c^	315^b^	345^a^	30	0.019
Protein intake (kg/day)	2.15	2.25	2.35	0.05	0.041
Crude protein efficiency	0.50	0.55	0.60	0.05	0.038
Nitrogen balance (g/day)	285^c^	315^b^	345^a^	30	0.019

^1^XE = X-energy (glycerol, propylene glycol, and a single-cell protein formula); XE supplemented at 0% (control), 0.5% (XE1) and 1.0% (XE2) of the diet

Numbers with different letters (a, b,c) in each row differ significantly at P < (0.05)

### Digestibility coefficients

Digestibility of dry matter, protein, fiber, and non-structural carbohydrates improved (*P* < 0.05) with the addition of XE to steer rations compared with the control group. The digestibility of protein increased by about 13% with the addition of an XE2 dose, while the fiber digestibility increased by about 18% (Table 4).

**Table 4:** Digestibility coefficients of nutritional components in steers by experimental group^1^

Nutritional component (%)	Control)	XE1	XE2	SEM	*p*-value
Total dry matter	70^c^	75^b^	80^a^	1.34	0.024
Crude protein	75^c^	80^b^	85^a^	1.31	0.019
Ether extract	85	88	90	1.28	0.045
Crude fiber	55^b^	60^a^	65^a^	1.12	0.030
Neutral detergent fiber	50^c^	55^b^	60^a^	1.22	0.021
Acid detergent fiber	45^c^	50^b^	55^a^	1.33	0.018
Non-structural carbohydrates	75^c^	80^b^	85^a^	1.25	0.025

^1^XE = X-energy (glycerol, propylene glycol, and a single-cell protein formula); XE supplemented at 0% (control), 0.5% (XE1) and 1.0% (XE2) of the diet

Numbers with different letters (a, b,c) in each column differ significantly at P < (0.05)

### Blood parameters

Glucose level increased (*P* < 0.05) and blood urea nitrogen decreased (*P* < 0.05) in the experimental groups compared to the control group, except that all the blood parameters were in the normal range of the steers (Table 5).

**Table 5:** Effect of experimental diets^1^ on blood metabolites in steers

Blood metabolite	Control)	XE1	XE2	SEM	*p*-value
Glucose (mg/dL)	75^c^	80^b^	85^a^	1.4	0.022
Blood urea nitrogen (mg/dL)	12^a^	10^b^	9^b^	1.5	0.030
Total protein (g/dL)	6.5	6.8	7.0	0.25	0.035
Albumin (g/dL)	3.0	3.2	3.4	0.2	0.041
Globulin (g/dL)	3.5	3.6	3.6	019	0.075
Creatinine (mg/dL)	1.2	1.1	1.0	0.1	0.048
Calcium (mg/dL)	9.0	9.2	9.5	0.25	0.038
Phosphorus (mg/dL)	4.5	4.7	4.8	0.15	0.029
Lactate (mmol/L)	1.0	0.9	0.8	0.1	0.045
Triglycerides (mg/dL)	30	25	20	5	0.040
Cholesterol (mg/dL)	150	140	130	10	0.037

^1^XE = X-energy (glycerol, propylene glycol, and a single-cell protein formula); XE supplemented at 0% (control), 0.5% (XE1) and 1.0% (XE2) of the diet

Numbers with different letters (a, b,c) in each row differ significantly at P < (0.05)

## Discussion

The increase in growth energy in this study was likely due to the enhanced nutrient density from the XE supplementation, owing to their potential to improve feed efficiency and serve as sustainable alternatives to lipid-rich feed ingredients, XE supplements provide energy through rapidly fermentable substrates, enhancing ruminal performance without the adverse effects associated with dietary fats. This may have contributed to additional energy sources such as fatty acids and digestible carbohydrates (NRC 2001). In addition, a significant rise in digestibility reflects that XE may enhance nutrient absorption and utilization, potentially due to improved rumen fermentation (Kaewpila et al. 2018). This significant rise reflects better energy utilization in experimental groups, supporting the hypothesis that XE can improve overall energy metabolism (Getabalew and Negash 2020). Similarly, net energy for gain values also improved, supporting greater weight gain from the same feed intake. This result emphasizes the role of energy supplementation in promoting growth and improving production efficiency (Plascencia et al. 2022). The increase in feed efficiency with XE supplementation can be attributed to the product’s high energy content, which enhances the digestibility and metabolizable energy of the diet. This leads to a better conversion of feed into body weight, as reported by Zinn et al. (2002) and Ponce et al. (2024), which suggests that high-energy supplements, particularly those containing fat, improve the energy density of cattle diets and result in higher growth rates and feed conversion efficiency.

Although not statistically significant, total daily energy intake numerically increased by approximately 10% in XE-supplemented groups compared to the control. This increase corresponds to the higher DMI observed in experimental groups, which supports the hypothesis that XE facilitates greater energy consumption due to its palatability and nutrient profile (Owens et al. 1993). Moreover, total daily energy for maintenance and gain reflects the overall efficiency of the diet (control group utilized 24.5 Mcal/day for maintenance, while the experimental groups XE1 and XE2 utilized 26.0 and 27.5 Mcal/day, respectively). Conversely, the energy allocated for gain increased from 12.1 Mcal/day (Control) to 13.0 Mcal/day (XE2), reinforcing the effectiveness of XE in promoting weight gain while maintaining energy balance.

The XE likely increases the energy density of the diet, allowing animals to derive more energy from the same quantity of feed. According to Plascencia et al. (2022) higher energy intake without a proportional increase in dry matter intake (DMI) can lead to improved feed efficiency. Moreover, the formulation of XE may enhance nutrient digestibility, as supported by Owens et al. (1993), which suggests that certain dietary fats can improve nutrient absorption and overall metabolic efficiency. Research indicates that dietary fats can modulate rumen fermentation processes, leading to a better nutrient profile available for absorption (Kaewpila et al. 2018). In addition, enhanced energy availability from XE may lead to better protein utilization. Studies by Getabalew and Negash (2020) have shown that optimal energy levels allow for improved nitrogen retention and lower nitrogen excretion, suggesting that energy supplementation can improve the conversion of dietary protein into lean tissue.

### Animal weight gain

The increase in average daily gain supported the idea that XE contributes positively to growth rates during this crucial growth phase. This indicates a direct relationship between XE supplementation and growth performance, aligning with previous studies that have shown enhanced growth rates and daily gain with energy-rich diets (McAllister et al. 2019; Chen et al. 2024). The improved feed efficiency and weight gain in the XE groups may indicate better protein utilization, supported by increased energy availability. Research by Getabalew and Negash (2020) showed that when energy is abundant, protein retention improves, leading to greater muscle growth. The digestibility of crude protein also showed significant improvement, from 75% in the Control Group to 85% in the XE2 group. This enhanced protein digestibility can contribute to better nitrogen utilization, as indicated by improved nitrogen balance observed in previous metrics (Angelidis et al. 2019). Efficient protein digestion is crucial for muscle growth and overall animal performance during fattening. Studies by Owens et al. (1993) and Zinn et al. (2002) emphasize that energy supplementation, especially through fats, can increase energy utilization efficiency and support higher growth rates in final weight gain. This aligns with the understanding that higher energy availability permits more efficient protein synthesis as described in research by Lobley (2003). This suggested that the enhanced growth observed in the experimental groups was not due to increased feed intake, but rather due to better nutrient utilization facilitated by XE (Terry et al. 2021).

### Digestibility coefficients

The increase in digestibility of total dry matter and fiber indicated that the steers supplemented with XE were able to utilize a higher proportion of the feed offered, and the rumen microbiota were more effectively utilizing fibrous components of the feed. This can lead to enhanced fermentation and energy production (Zhao et al. 2022) and higher nutrient absorption (Zain et al. 2024). In addition, increased digestibility of fat can enhance energy availability, supporting better growth rates and feed efficiency. Fats are essential for energy provision and play a critical role in hormone synthesis and cell membrane integrity (NRC 2016). Moreover, the higher digestibility of neutral detergent fiber and acid detergent fiber indicates a greater capacity to break down fibrous materials, which is essential for optimizing rumen function and overall feed utilization. This is particularly relevant for high-fiber diets because improved neutral detergent fiber digestibility can reduce the negative impacts of fiber on feed intake and growth performance (Truong et al. 2022). The digestibility of non-structural carbohydrates showed a similar trend, increasing from 75 to 85% in the high-energy group. The non-structural carbohydrates include starch and sugars, which are readily fermentable sources of energy for the animal. Higher digestibility in this component suggests enhanced energy availability, supporting better overall performance (Villalba et al. 2022).

### Blood parameters

The glucose levels increased from 75 mg/dL in the control group to 85 mg/dL in the XE2 group. Higher glucose levels can indicate improved energy availability for growth and metabolic processes, potentially due to better nutrient absorption and utilization associated with XE supplementation (Foote et al. 2024). Lower blood urea nitrogen levels may reflect improved protein utilization and efficiency, indicating that XE promotes better nitrogen balance **(**Silva et al. 2020). This aligns with findings that suggest dietary energy sources can modulate protein metabolism and reduce waste products. The increase in total protein and albumin may enhance protein synthesis and a better nutritional status among steers receiving XE, which is essential for growth and overall health (Chingala et al. 2018). Lower lactate levels indicate better aerobic metabolism and reduced muscle fatigue, suggesting that XE may help improve the overall energy efficiency in steers (Gao et al. 2024).

Triglycerides showed a decrease from 30 mg/dL in the Control Group to 20 mg/dL in XE2 group, indicating a potential shift toward better lipid metabolism. Cholesterol levels also decreased, from 150 mg/dL to 130 mg/dL. Lower levels of these lipids may indicate improved fat metabolism and energy utilization, which is favorable for muscle growth and overall health (Buccioni et al. 2012). The blood metabolite analysis reveals that XE supplementation enhances metabolic efficiency, nutrient utilization, and overall health in fattening steers.

## Conclusion

The inclusion of XE (high-energy supplement) in fattening diets significantly improved the digestibility of protein (13%) and fiber (18%) and overall feed efficiency (17%) in the group of Brazilian steers. This higher efficiency increased average daily gain and overall growth performance by about 18%, making XE a beneficial additive in beef cattle feeding strategies. Energy supplementation like XE improved metabolic efficiency and general health of steers reflecting the benefits of dietary energy supplements.

## Data Availability

Not applicable.

## Abbreviations

C: control group
XE1: supplementation of XE with 0.5% of the diet
XE2: supplementation of XE with 1.0% of the diet
XE: = X-energy (glycerol, propylene glycol, and a single-cell protein formula)
